# Comparison of Vaginal Birth Rate between Induction of Labour and Expectant Management at 40 Weeks in Women with a Previous Caesarean Section: A Pilot Randomized Controlled Trial

**DOI:** 10.1155/2023/9189792

**Published:** 2023-08-21

**Authors:** K. Rajalakshmi, Gowri Dorairajan, Swetha S. Kumar, C. Palnivel

**Affiliations:** ^1^Department of Obstetrics and Gynecology, Jawaharlal Institute of Postgraduate Medical Education and Research (JIPMER), Puducherry, India; ^2^Department of Preventive and Social Medicine, JIPMER, Puducherry, India; ^3^Department of Community Medicine, Jawaharlal Institute of Postgraduate Medical Education and Research (JIPMER), Puducherry, India

## Abstract

**Background:**

The optimum time of labour induction among women with a previous caesarean without any pregnancy complication and eligible and willing for vaginal delivery is not specified. This study compares the vaginal birth rates between induction at 40 weeks and expectant management till 41 weeks.

**Method:**

We conducted this parallel design nonblinded, randomized controlled trial in a tertiary care teaching institution in South India on women with a previous lower segment caesarean section eligible for a trial of labour with singleton foetus without any pregnancy complication at recruitment. We screened 1886 women. Sixty women underwent block (of 6 each) randomization into two groups of thirty each at 40 weeks. We induced the women in the intervention group at 40 weeks with oxytocin or a single 24-hour application of a Foley catheter followed by oxytocin infusion and amniotomy. The expectant group underwent maternal and foetal surveillance and induction at 41 weeks with the same protocol if not delivered by then. We compared the primary outcome of the proportion of vaginal birth rate with a chi-square test.

**Result:**

Data from all sixty women were analyzed. Twenty (66.67%) in the induction compared to ten (33.33%) in the expectant group delivered vaginally. This difference was significant (RR 2.0, 95% CI: 1.13-3.52; *P* = 0.016). One woman in the expectant group had scar dehiscence.

**Conclusion:**

Among women with a previous caesarean scar, labour induction at 40 weeks has a significantly higher vaginal birth rate than those managed expectantly till 41 weeks. More extensive trials are feasible and recommended. *Trial Registry*. The trial was prospectively registered with the clinical trial registry of India. This trial is registered with CTRI/2018/09/015719 (date of registration 14th September 2018).

## 1. Introduction

The optimal delivery timing for women with a previous caesarean section without maternal or foetal complications is not well studied. Obstetric Societies recommend a labour after caesarean section (TOLAC) for all eligible cases, and guidelines are available for eligibility for TOLAC [1–3]. The Royal College of Obstetricians and Gynaecologists (RCOG) recommends 41 weeks to terminate these eligible patients if they do not go into spontaneous labour by the expected delivery date [1]. Spontaneous labour is considered safer and has a higher chance of successful vaginal birth after caesarean (VBAC) than induced. Studies have shown a one-and-a-half times higher risk of caesarean section (CS) and a two to three times higher risk of rupturing the uterus with the induction of labour (IOL) than spontaneous labour [4–7]. Awaiting beyond 40 weeks hopes they will achieve spontaneous labour. However, past dates have problems [8, 9], like meconium passage and amniotic fluid reduction. Recently published meta-analysis [10] also concluded that there is a 64% increased risk of stillbirth on continuing pregnancies to 41 weeks instead of delivery at 40 weeks. The most extensive retrospective study on 46,176 women with previous caesarean delivery has shown that IOL at 39 weeks was found to have lower odds of CS compared to those managed expectantly [11]. Successful and safe vaginal births will be an asset in limiting the escalation in repeat caesarean section rates. Expectant management and not spontaneous labour is an accurate comparator for elective IOL at term. To our knowledge, no randomized controlled trials have compared expectant management with IOL. We undertook this pilot randomized controlled trial to compare the successful vaginal birth rates in women with previous one lower segment CS when induced at 40 weeks compared to expectant management till 41 weeks.

## 2. Methods

### 2.1. Design and Settings

This parallel-arm design randomized, nonblinded controlled trial was carried out in the antenatal ward and labour room of the Department of Obstetrics and Gynaecology at Women and Children Block of Jawaharlal Nehru Institute of Postgraduate Medical Education and Research (JIPMER) from September 2018 till June 2020 after obtaining approval from the institute ethics committee (JIP/IEC/2018/0145) (available at http://www.ctri.nic.in/Clinicaltrials/pdf_generate.php?trialid=27789&EncHid=&modid=&compid=%27,%2727789det%27). JIPMER is a tertiary care teaching hospital located in South India.

### 2.2. Eligibility Criteria

We included women over 18 years, with previous single lower segment caesarean delivery at 40 weeks with a singleton foetus in vertex presentation, no pregnancy complications, and eligible for TOLAC. Women with interpregnancy interval greater than 18 months, scan estimated foetal weight less than 3.5 kg, nonrecurrent indication for the previous section, the previous scar restricted to lower segment, scar thickness more than 3 mm on ultrasonography, and no clinical evidence of cephalopelvic disproportion were considered eligible for TOLAC. The previous caesarean carried out for placenta previa, transverse lie, preterm and late in the second stage of labour, or clinical evidence of uterocervical infections after the previous caesarean section was considered ineligible for TOLAC as these indications have high clinical suspicion for an extension to upper segment fibres/poor strength due to infection and/or micro hematoma formation.

### 2.3. Exclusion Criteria

We excluded women with complications like preeclampsia, diabetes mellitus, oligohydramnios, or intrauterine growth restriction (IUGR), foetal malformations, and multiple gestations, those who are not willing for the trial of labour after caesarean, women requiring IOL for any other maternal or foetal condition before randomization, and women with an unestablished period of gestation.

### 2.4. Primary Outcome

The primary outcome is the successful vaginal birth rates in the two groups.

### 2.5. Secondary Outcome(s)

The secondary outcomes are as follows: (i) maternal complications like scar rupture, postpartum haemorrhage, sepsis, and any mortality and (ii) perinatal outcomes like birth asphyxia, respiratory morbidities, hyperbilirubinemia, sepsis, and perinatal mortality.

There were no changes in the outcomes after the trial commenced.

### 2.6. Sample Size Calculation

VBAC rate with spontaneous labour varies from 72% to 85%, depending on the parity [1]. VBAC rates reported with the Foley induction vary from 18% [12] to 71% [13]. We presumed that all women in the expectant arm go into spontaneous labour, and presuming the VBAC rate of 79% for spontaneous labour for a mixed parity population and VBAC rate of IOL with Foley to be 40% [14], we needed thirty cases each in both groups for an 80% power with 95% confidence.

### 2.7. Randomization Details

We carried out block randomization (block size of 6 each) using varying numbers generated via computer using randomization software. The allocation ratio was 1 : 1. Allocation concealment was done by someone unrelated to the study from the Department of Preventive and Social Medicine through serially numbered opaque sealed envelopes. The principal investigator enrolled the participants. There was no blinding.

### 2.8. Study Procedure

Women with uncomplicated pregnancies with a previous caesarean section were screened from 39 weeks onwards clinically and with sonography for eligibility for TOLAC. The gestation period was calculated as per the last menstrual period (LMP) and was corrected based on the crown-rump length scan of 14–41 mm if the discrepancy in the expected delivery date was ≥7 days from that based on the LMP. After obtaining informed written consent, we randomized the women fulfilling the study criteria and willing for TOLAC to either group on the day of completion of 40 weeks. We reassessed the Bishop score after randomization.

We induced women in the induction group (group II) on the same day (40 weeks) or within 24 hours of randomization. If the Bishop scored unfavourable (<6), induction was done by ripening the cervix with a single application of 22 French Foley single balloon catheter inflated to 60 ml just above the internal os, under aseptic precautions. The women were monitored for onset of contractions/leaking/bleeding/any other complication and the foetus by intermittent auscultation and a nonstress test after 12 hours. The Foley catheter was deflated and removed 24 hours later if she did not spontaneously expel it or earlier if she developed a spontaneous rupture of membranes. We noted the Bishop score again and started low-dose oxytocin infusion delivered through a pump starting from 3 mIU/min and incremented by 3 mIU every half-hourly until she achieved good contractions or a maximum of 24 mIU/min. We performed an artificial rupture of membranes once the women started regular contractions or after four hours of the maximum dose of oxytocin. We used an electronic tocodynamometer to monitor the foetal heart and uterine contractions. We monitored the labour progress and symptoms and signs of scar dehiscence. If women failed to achieve active labour (4 cm dilatation with 75-100% effacement), even after 6 hours of maximum dose of oxytocin or 6 hours of good uterine contractions, it was considered as failed induction and terminated by caesarean section. The trial of labour continued if she achieved active labour.

Women in the expectant arm of the study remained admitted to the hospital as a standard policy. We monitored the foetus by a nonstress test (NST) every 48 hours and a repeat ultrasound for amniotic fluid index and biophysical profile for foetal surveillance at 40 weeks plus three days. It was repeated at 41 weeks if she had not delivered by then. In case any complications arose between 40 and 41 weeks, pregnancies were terminated either by IOL or by caesarean as per the case. W carried out IOL in them as per the protocol followed in the induction group if not already delivered by 41 weeks. We followed up with the neonates and women till discharge from the hospital. We did not change the inclusion criteria or methodology after trial commencement.

### 2.9. Statistical Analysis

We expressed continuous variables as mean with standard deviation or median with interquartile range and summarized categorical data as frequency, percentage, or proportion. The primary outcome variable (mode of delivery) was expressed as the proportion with a 95% confidence interval and was analyzed using the chi-square test as the statistical test. We compared the baseline characteristics using the chi-square test or Fischer's exact test if expressed in proportions and the unpaired student test or Mann–Whitney test. Subgroup analysis was attempted for factors associated with successful VBAC after IOL by univariate and logistic regression analyses.

## 3. Results

**Figure 1:**
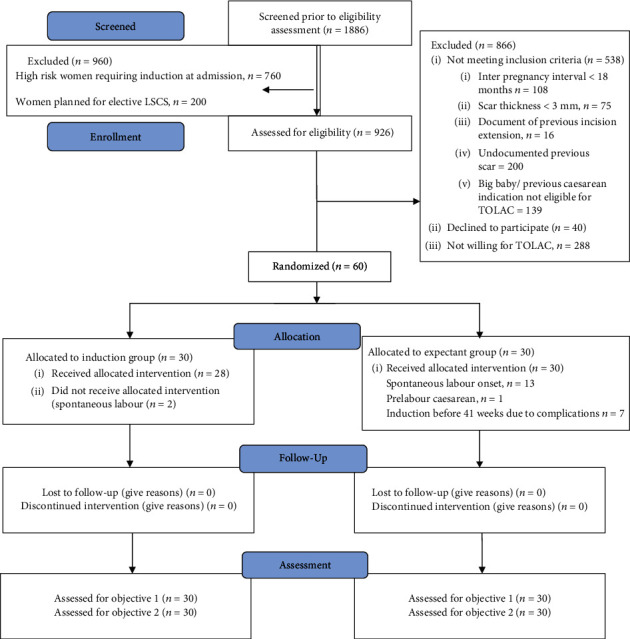
Consort diagram.

We screened 1886 women; 966 were otherwise low-risk and could undergo expectant management. 35% of these were either unwilling for TOLAC or the study. Sixty women fulfilled the inclusion criteria (Figure 1). All 60 recruited patients (30 in each group) completed the study. Two patients in the induction arm underwent spontaneous labour after randomization before induction on the expected delivery date. One woman in the expectant arm had a spontaneous version to breech presentation at 41 weeks, so we did a prelabour caesarean to deliver her. We performed intention-to-treat analysis and did not exclude these three cases from the analysis.

Table 1 shows the demographic and pregnancy-related variables. The mean age, socioeconomic status (modified BG Prasad classification [15]), parity, previous history of VBAC, indication of the previous caesarean, and preinduction Bishop score were comparable in the two groups.

There was a significantly higher VBAC rate in the induction group. Twenty of the 30 (66.67%) women in the induction group had a successful vaginal birth. Sixteen had a vaginal delivery, and four had an instrumental vaginal delivery (vacuum-assisted delivery). For varied indications, the remaining ten subjects (33.33%) underwent caesarean section after the onset of labour. The expectant group had a VBAC rate of 33.33%, with only 10 out of the 30 women undergoing successful vaginal delivery, and this difference was significant (RR 2.0, 95% CI: 1.13-3.52; *P* = 0.016).

In the expectant group, out of the 30 women, thirteen (43.33%) went into spontaneous labour before 41 weeks (seven of them had VBAC (54%)), 7 underwent induction before 41 weeks for different complications (one developed gestational hypertension, five had oligohydramnios without premature rupture of membranes, and one had reduced foetal movements), and nine underwent induction at 41 weeks as per protocol. One had a prelabour CS for the spontaneous conversion to breech presentation (Table 2).  Table 3 shows the intrapartum details. The proportion of women with abnormal foetal heart rate patterns and meconium-stained liquor and the duration of oxytocin infusion were comparable in the two groups. The birth weight was higher by 270 grams in the expectant group. This difference was statistically significant though not clinically significant. The caesarean section rate was significantly higher in the expectant group compared to the induction group (chi‐square = 6.67, df = 1, *P* = 0.01). The indication for CS in the two was not significantly different (Table 4).

One case of scar dehiscence in the expectant arm was confirmed intraoperatively. This woman did not go into spontaneous labour during the expectant management period. She underwent labour induction at 41 weeks as per protocol. She underwent an emergency caesarean for failed induction. However, intraoperatively, a 3 cm scar dehiscence was observed. We repaired the rent, and the woman recovered uneventfully. There were no cases of scar dehiscence in the induction at 40 weeks arm. There was no third or fourth degree perineal tear in women with spontaneous or operative vaginal delivery.

None of the women developed any complications like sepsis or postpartum haemorrhage. None of them required a blood transfusion or hysterectomy.

There were no stillbirths. None of the babies were born with a low Apgar score. None of the neonates required admission to an intensive care unit. There was no morbidity or mortality among these sixty newborns. We could not assess the perinatal and maternal outcomes as our study did not have the power for it. We recommend future trials powered for these outcomes.

## 4. Discussion

In our pilot randomized controlled trial, we found that in the uncomplicated pregnancies with a previous caesarean delivery, IOL at 40 completed weeks achieved significantly higher successful vaginal deliveries than planned expectant management till 41 weeks. Our study was not powered to analyze maternal or perinatal outcomes. We had strict inclusion criteria to reduce the possible rates of scar dehiscence. So, we excluded many women from the trial who could have undergone TOLAC if they were in spontaneous labour. However, 13 (43%) of the women in the expectant group in our study went into spontaneous labour. Seven of these thirteen (54%) delivered vaginally. Another seven women (23%) in the expectant group developed a maternal or foetal complication needing induction before 41 weeks (one woman (3.3%) developed gestational hypertension de novo). The VBAC rates among those induced after 40 weeks in the expectant arm were much lower (19%) than those who had spontaneous labour (54%). The VBAC rate of spontaneous labour after 40 weeks compares well with those induced before 40 weeks.

The landmark ARRIVE trial [16] and the two meta-analysis [17, 18] of randomized controlled trials have shown a reduction in the adverse perinatal outcome and caesarean delivery rates in those undergoing IOL at 39 weeks compared to those managed expectantly—however, all these involved women without a previous caesarean section. The meta-analysis of the cohort study by Wood et al. [19] also reported similar results. None of the 6 cohort studies they analyzed included women with a previous caesarean section.

To our knowledge, there are no randomized controlled trials on this subject in women with previous caesarean delivery. Only a few cohort studies are comparing IOL with expectant management in women with a previous caesarean section at term. The first study was a large retrospective cohort of 16 years [11]. It showed lower odds of caesarean and higher odds of spontaneous vaginal delivery in the IOL group at various gestation periods from 39 to 41 weeks. They did not find a higher rate of rupture in the IOL group. However, heterogeneity and changing practices over the 16-year study could have influenced the results. In another study [20], the authors reported higher VBAC rates of 73.8% vs. 61.3% among the induced versus those managed expectantly at 39 weeks (*P* < 0.001) (OR1.3), but they also found an increased rate of rupture in the IOL group. The study was a secondary analysis of a registry from a 4-year multicentre observational study [21]. The original study has not detailed the induction protocol. Many women had received prostaglandins with or without oxytocin, and information on confounder-like cervical status in the two groups was unavailable.

In another secondary analysis study of an obstetric cohort from the consortium of safe labour, among women with previous caesarean section who attempted TOLAC, the authors [22] observed higher rates of failed VBAC in the women induced from 37 to 39 weeks but not at 40 weeks. Their study cohort included both high- and low-risk women, which could have influenced a quicker decision to terminate labour by emergency caesarean section.

A 3-year retrospective cohort was recently published [23]. The authors observed that IOL at 39 weeks was associated with lower intra-amniotic infection among women with previous caesarean section and improved 5-minute APGAR score in the IOL arm but at the cost of a 70% increase in caesarean delivery rates. They also observed that 2.2% of the expectant arm women developed new-onset hypertension after 39 weeks. In this study, the induction protocol was not detailed, and the authors did not exclude oligohydramnios as an indication for induction. Also, the authors excluded women who did not go into spontaneous labour in the expectant arm but opted for scheduled caesarean section instead of IOL. This fact could have influenced the higher caesarean rate reported in the induction arm compared to the expectant arm.

In our study, we had strict inclusion criteria. We took 3 mm as the cut-off for eligibility for TOLAC though RCOG recommends scar thickness above 2 mm. We took a higher cut-off for the total thickness of the scar on sonography because IOL has a 2 to 3 times higher risk of scar rupture, and the meta-analysis done in 2013 [24] had shown variable sensitivity for variable cut-offs and that the total thickness more than 3 mm provided the most substantial negative predictive value of occurrence of defect during TOLAC. We used a Foley single balloon catheter to ripen the cervix, which is considered a safe method in women with previous caesarean sections. We used oxytocin infusion followed by artificial rupture of membranes in those with the Bishop score less than six despite one application of the Foley balloon ripening. We found that such a trial is feasible and can be more pragmatic in the future.

### 4.1. Strengths and Limitations

Our study is a pilot randomized controlled trial. The two groups were comparable for essential variables like the Bishop score and previous vaginal delivery that are known to influence VBAC rates. We had a strict induction protocol for both groups. We excluded any maternal or foetal complications requiring induction, including oligohydramnios at the time of recruitment at 40 weeks. Since we followed standard induction protocol and monitoring, we can generalise the results to most tertiary centres with infrastructure for delivering women with previous caesarean pregnancy. There can be potential bias as there was no blinding. The limitation of our study is as follows: the sample size was small and could not have detected any harm. We did not power the sample size for perinatal or maternal outcomes. To calculate the sample size, we presumed that all participants in the expectant group are likely to have spontaneous labour, but this may not be the reality always. We terminated labour by caesarean section if the women failed to enter the active phase of labour within 6-8 hours of artificial rupture of membranes. We took 4 cm dilation as the definition for the active phase of labour. The trial of labour did not last longer than 24 hours if the women had not gone into the active phase of labour. We did not give a second application of the Foley if the Bishop score had not improved. However, since the induction protocol was the same for both groups, this definition of active labour and failed induction would not have influenced the difference in the vaginal delivery rates.

### 4.2. Interpretation

An RCT comparing IOL at 40 weeks with expectant management in women with previous caesarean sections is feasible and acceptable. We need to screen a large population for more extensive trials powered to detect any harm or difference in perinatal outcomes. Even with a small sample size of 30 in each arm, we found a significantly higher VBAC rate in the IOL group. Only 43% of those in expectant management underwent spontaneous labour.

## 5. Conclusion

We recommend more extensive trials to study the safety and maternal and perinatal outcomes of induction at 40 weeks versus expectant management up to 41 weeks for women with singleton foetus with a previous caesarean scar who are eligible for TOLAC.

## Figures and Tables

**Table 1:** Demographic and pregnancy characteristics in the two groups.

Parameters		Group I (expectant) (*N* = 30)	Group II (induction) (*N* = 30)	*P* value
Age (years)	21-25	46.67%	46.67%	*X* ^2^ = 0.182, *P* = 0.91
	26-30	43.33%	40%	
	31-35	10%	13.33%	

Socioeconomic status	Upper middle	6.67%	6.67%	*X* ^2^ = 5.46, *P* = 0.14
	Lower middle	20%	3.33%	
	Upper lower	36.67%	60%	
	Lower	36.67%	30%	

Parity	Primiparous	90%	86.67%	*X* ^2^ = 5.2, *P* = 0.50
	Multiparous	10%	13.33%	

Occupation	Housewife	63.33%	73.33%	*X* ^2^ = 01.55, *P* = 0.67
	Daily waged	23.33%	16.67%	
	Monthly waged	10%	10%	
	Business	3.33%	0	

Previous VBAC^a^		3.33%	3.33%	*P* = 1
Mean age (years)		26.13 ± 3.41	26.4 ± 3.8	
Bishop score at 40 weeks (mean ± SD^b^)		3.07 ± 1.11	3.5 ± 1.17	*t* = −1.47, *P* = 0.07
Interpregnancy interval in months (mean ± SD^b^)		36.07 ± 17	38.87 ± 20.64	*t* = −0.5, *P* = 0.57
Scar thickness in mm (mean ± SD^b^)		3.95 ± 0.25	4 ± 0.24	*t* = −0.86, *P* = 0.39
AFI^c^ (mean ± SD^b^)		10.08 ± 2.53	10.31 ± 2.48	*t* = −0.35, *P* = 0.72

^a^VBAC: vaginal birth after caesarean; ^b^SD: standard deviation; ^c^AFI: amniotic fluid index; *X*^2^: chi-square; *t*: Student test.

**Table 2:** Mode of delivery for women in the two groups.

Group		*N* (%)	VBAC (*N*, %)	Emergency LSCS	*P* value
Induction (group II)	Per protocol	30	20 (66%)	10	*X* ^2^ = 6.67, *P* = 0.01^∗^
Expectant (group I)	IOL^a^	30	10 (33.3%)	20	
	Per protocol at 41 weeks	9 (30%)	2	7
	Between 40 and 41 weeks	7 (3.33%)	1	7
	Spontaneous onset of labour	13 (43.33%)	7 (54%)	6	
	Prelabour caesarean section	1 (3.33%)	—	—	

^a^IOL: induction of labour. ^∗^ - significant.

**Table 3:** Comparison of intrapartum details between the two groups.

Parameter	Group I (expectant) (*N* = 30)	Group II (induction) (*N* = 30)	*P* value
Birth weight (mean ± SD^a^)	3.295 ± 0.304	3.022 ± 0.348	*t* = 3.24, *P* = 0.002^∗^
Duration of oxytocin infusion (hours) (mean ± SD^a^)	7.76 ± 4.63	6.92 ± 3.81	*P* = 0.511
Meconium-stained liquor, *n* (%)	8 (26.67%)	6 (20%)	*P* = 0.542
Foetal heart rate pattern, *n* (%)			
Early deceleration	2 (6.67%)	4 (13.33%)	*P* = 0.861
Late deceleration	1 (3.33%)	1 (3.33%)	
Variable deceleration	2 (6.67%)	2 (6.67%)	

^a^SD: standard deviation; *t*: Student *t*-test.  ^∗^ - significant.

**Table 4:** Comparison of indications for emergency caesarean section in present pregnancy between the two groups.

Indication (*N*, %)	Group I (*N* = 20)	Group II (*N* = 10)	*P* value
Failed induction	9 (45%)	4 (40%)	0.8
NPOL^a^	2 (10%)	0	
Foetal distress	4 (20%)	4 (40%)	0.2
CPD^b^ diagnosed in labour	0	1 (10%)	
Suspected scar dehiscence	3 (15%)	0	
Meconium-stained liquor with unfavourable cervix	1 (5%)	1 (10%)	0.6
Spontaneous conversion to breech	1 (5%)	0	

^a^NPOL = nonprogress of labour; ^b^CPD = cephalopelvic disproportion.

## Data Availability

The data will be provided on reasonable request.
